# The Effect of Electrolytic Hydrogenation on Mechanical Properties of T92 Steel Weldments under Different PWHT Conditions

**DOI:** 10.3390/ma13163653

**Published:** 2020-08-18

**Authors:** Lucia Čiripová, Ladislav Falat, Viera Homolová, Miroslav Džupon, Róbert Džunda, Ivo Dlouhý

**Affiliations:** 1 Institute of Materials Research, Slovak Academy of Sciences, Watsonova 47, 04001 Košice, Slovakia; lciripova@saske.sk (L.Č.); vhomolova@saske.sk (V.H.); mdzupon@saske.sk (M.D.); rdzunda@saske.sk (R.D.); 2 Institute of Physics of Materials, CEITEC-IPM, Czech Academy of Sciences, Zizkova 22, 61662 Brno, Czech Republic; idlouhy@ipm.cz

**Keywords:** grade 92 steel weldment, post-welding heat treatment, tensile straining, hydrogen embrittlement, metallography and fractography

## Abstract

In the present work, the effects of electrolytic hydrogen charging of T92 steel weldments on their room-temperature tensile properties were investigated. Two circumferential weldments between the T92 grade tubes were produced by gas tungsten arc welding using the matching Thermanit MTS 616 filler material. The produced weldments were individually subjected to considerably differing post-welding heat treatment (PWHT) procedures. The first-produced weldment was conventionally tempered (i.e., short-term annealed below the Ac_1_ critical transformation temperature of the T92 steel), whereas the second one was subjected to its full renormalization (i.e., appropriate reaustenitization well above the T92 steel Ac_3_ critical transformation temperature and subsequent air cooling), followed by its conventional subcritical tempering. From both weldments, cylindrical tensile specimens of cross-weld configuration were machined. The room-temperature tensile tests were performed for the individual welds’ PWHT states in both hydrogen-free and electrolytically hydrogen-charged conditions. The results indicated higher hydrogen embrittlement susceptibility for the renormalized-and-tempered weldments, compared to the conventionally tempered ones. The obtained findings were correlated with performed microstructural and fractographic observations.

## 1. Introduction

The 9 wt.% Cr creep strength enhanced ferritic (CSEF) steels (e.g., T/P91, T/P92, T/P911, C/FB2, MARBN, NPM1, etc.) represent advanced structural materials for application in high-efficiency power engineering. However, for constructing complex power generation equipment, fusion welding technologies are needed for joining individual functional parts. In accordance with the numerous research studies and ex-service experience, e.g., [1,2,3,4,5], it has been generally accepted that the fusion welded joints of ferritic steels represent the most critical component locations with respect to their preferential degradation and potential failure. Besides the regions of base material (BM) and weld metal (WM) within the structures of all welded joints, thermal effect of fusion welding on the welded ferritic steels’ BMs typically results in the creation of a relatively wide heat-affected zone (HAZ) consisting of several, continuously created microstructural sub-regions, i.e., often called the “HAZ microstructural gradient”. Its occurrence within the welded joint represents the primary, welding-induced microstructure degradation zone, since the individual HAZ sub-regions, such as the coarse-grained HAZ (CG-HAZ), fine-grained HAZ (FG-HAZ), inter-critical HAZ (IC-HAZ), and subcritical HAZ (SC-HAZ), possess mutually various microstructures and mechanical properties [6,7,8]. 

Depending on several factors including the welding metallurgy-related material properties and outer loading and/or environmental conditions, the fusion weldments can be susceptible to some of several typical failures [9]. The “Type I” and “Type II” failures, originating from intercrystalline cracks in weld metals, are generally related to the so-called “hot cracking” phenomena, typically occurring in weld solidified microstructures with higher impurity content. However, the occurrence of these failures has been considerably suppressed in the ferritic steels’ weldments thanks to the recently developed ferritic filler materials of high metallurgical purity [10]. The “Type III” failure typically occurs within the CG-HAZ close to the weld fusion zone (FZ) of low alloy ferritic steel weldments. This failure type has been often related to the so-called “reheat cracking” due to either residual stress relief during the PWHT or superabundant secondary precipitation hardening in FZ/CG-HAZ during high temperature creep exposure [11,12,13]. In a specific case of dissimilar weldments, the considered failure type (sometimes referred to as “Type IIIa” failure [14]) is related to premature creep cracking within the area of soft, carbon-depleted CG-HAZ, created as a result of the decarburization processes driven by the carbon activity gradient at the interface between the lower grade ferritic steel and the higher grade weld metal. Last but not least, depending on acting environmental conditions, the “Type III” failure may also be related to so-called “cold cracking” phenomena, i.e., hydrogen-induced cracking (HIC) or environmentally assisted cracking (EAC) [15,16]. This failure occurs due to exceeding the critical hydrogen concentration in locally hardened FZ/CG-HAZ areas with the highest degree of transformation (i.e., martensitic) hardening as a consequence of the welding thermal cycle. Under long-term creep conditions, the welded joints of ferritic heat-resistant steels are typically prone to the “Type IV” failure within their FG-/IC-HAZs because these regions exhibit the lowest creep strength within the whole weldment. This failure is generally related to severe degradation of transformation hardening mechanism and preferential coarsening of Fe_2_(W,Mo)-based Laves phase within the failure location [17,18]. The study by Albert et al. [19] showed that the “Type IV” failure is caused by preferential creep strain accumulation in the soft, fine-grained HAZ regions (FG-/IC-HAZs) due to the multiaxial stress state induced by microstructural heterogeneity throughout the weld-joint. A specific failure type is related to “cracking in over-tempered base material” which typically occurs within the softened region of SC-HAZ and is characterized by a highly ductile fracture [20,21]. This failure type is observed usually in welded joints after the high temperature tensile tests or after high-stress short-term creep tests [21,22]. The mechanism of microstructural and property degradation in SC-HAZ, i.e., within the over-tempered base material, is believed to arise from the coarsening of precipitates during the welding thermal cycle. After longer durations of low-stress creep tests, the failure commonly shifts from the over-tempered region to the “Type IV” failure region. However, unlike the short-term “over-tempered base metal cracking”, the long-term “type IV cracking” is characterized by low-ductility creep failure [23].

In common industrial practice, the weldments of CSEF steels are necessarily subjected to conventional PWHT, i.e., the subcritical tempering below the steel Ac1 critical transformation temperature. The main aim of such PWHT is to relieve residual stresses and thermally stabilize the weld microstructure with secondary phase precipitates, typically the M_23_C_6_ (M = Cr, Fe …) carbides and MX (M = V, Nb; X = C, N) carbo-nitrides. The direct consequence of performing the subcritical PWHT procedure is related to the decrease of unallowably high hardness in FZ/CG-HAZ and improvement of the overall weld fracture resistance [19,24]. However, it has been proved [19] that the occurrence of premature “Type IV” creep failure in ferritic steels’ weldments cannot be avoided by any variation in the subcritical PWHT regime, since their HAZ microstructural gradients remain still preserved within subcritically tempered microstructures. On the other hand, several studies [25,26] suggested that the only way to enable the “Type IV” failure suppression in ferritic welds is associated with so-called “full heat treatment” which involves the weld renormalization (i.e., the weld complete reaustenitization and its subsequent cooling on still air), followed by conventional subcritical tempering.

Our previous investigations [27,28] were focused on investigation of the effects of both the conventional tempering and quenching-and-tempering PWHT procedures of T92/TP316H martensitic/austenitic weldments on their microstructure and creep behavior. The results showed that the quenching-and-tempering PWHT led to “Type IV” failure elimination and thus notable creep life improvement as a result of significant homogenization of the T92 steel microstructure, i.e., complete suppression of the T92 HAZ microstructural gradient thanks to performed reaustenitization. Moreover, our separate study [29] on the T92 HAZ local mechanical properties of the T92/TP316H weldments indicated, that compared to the weldments subjected to only conventional PWHT, the T92 HAZ of quenched-and-tempered weldments exhibited lower hardness and higher impact toughness. The combined effects of quenching-and-tempering PWHT and subsequent electrochemical hydrogen charging on room-temperature tensile properties of the T92/TP316H weldments were investigated in [30]. It has been revealed that the applied electrochemical hydrogen charging did not affect the strength properties of the weldments significantly, but it resulted in quite serious deterioration of their deformation properties along with significant impact on their fracture behavior and final failure localization. The most critical region was found to be the interfacial weld region close to the T92 steel FZ. 

Our present study represents a continuous research work to our aforementioned former studies. It deals with investigation of the effects of initial PWHT conditions and subsequent electrochemical hydrogenation on the resulting room-temperature tensile properties and fracture behavior of T92/T92 welded joints. Mutual correlations between varying microstructural characteristics induced by different initial PWHT regimes and resulting mechanical properties of the weldments in either hydrogen-free or hydrogen-charged conditions are discussed.

## 2. Materials and Methods

Four segments of industrially normalized and tempered T92 tubes (outer diameter 38 mm, wall thickness 5.6 mm, approx. tube segment length 130 mm) were circumferentially welded in the company SES a.s. Tlmače, Slovakia. The welded joints were produced by gas tungsten arc welding (GTAW) technique using T92-based filler metal Thermanit MTS 616 to prepare two equivalent T92/T92 weldments. The T92/T92 welds geometry was the same as also used in our previous study about long-term ageing effects on room-temperature tensile behavior of quenched and tempered T92/TP316H dissimilar weldments [31]. Specifically, the 60° groove angle and 2–3 mm root gap was used. Welding parameters for the preparation of T92/T92 welded joints were the following ones: welding current 120–160 A, voltage 12–17 V and heat input 9–12 kJ/cm. The diameter of TIG electrode was 2.4 mm and the negative polarity on the electrode was used. Table 1 shows chemical compositions of the T92 steel base material (T92 BM) and T92 steel-based filler metal (T92 FM) Thermanit MTS 616.

The chemical compositions in Table 1 represent certified alloy compositions by the material producers Tenaris Dalmine (Dalmine—BG, Italy) and Voestalpine Böhler Welding (Düsseldorf, Germany), respectively.

**Figure 1:**
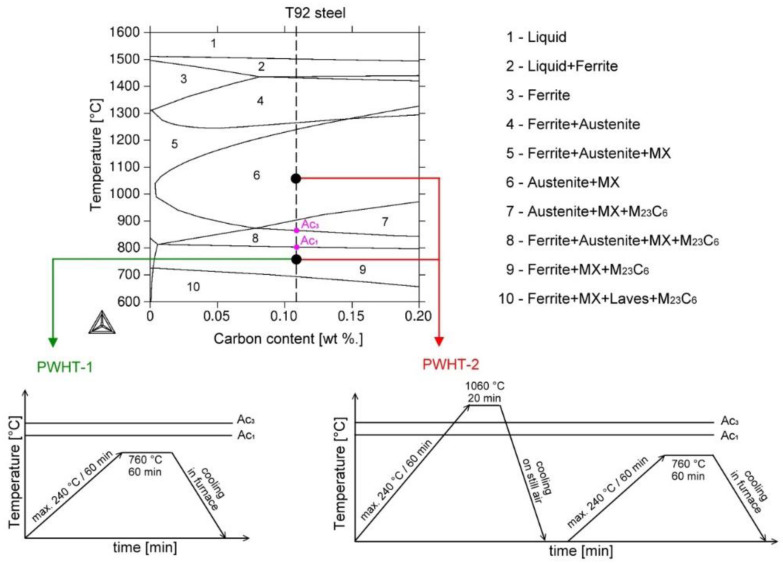
Calculated equilibrium phase diagram with schematic illustrations of individual post-welding heat treatment (PWHT) regimes applied in the present study for T92/T92 weldments (The T92 steel composition is indicated in the diagram by vertical dashed line at 0.11 wt.% C).

The two prepared weldments were individually subjected to mutually differing post-welding heat treatment (PWHT) procedures. Figure 1 shows schematic illustration of both these PWHT procedures in context with the equilibrium phase diagram including isoplethal section for T92 BM, computed by thermodynamic software ThermoCalc (version S, Thermo-Calc Software AB, Solna, Sweden) using thermodynamic database TCFE6.

**Figure 2:**
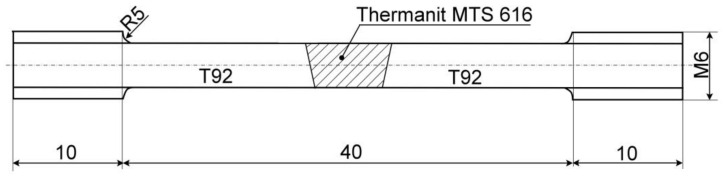
The tensile test specimen for cross-weld tensile testing of T92/T92 weldments (All dimensions are in mm), gauge length diameter 4 mm, gauge length 38 mm.

The first T92/T92 weldment was conventionally tempered at 760 °C (i.e., below the Ac_1_ temperature of T92 steel) for 60 min and then slowly cooled within the tempering furnace (see the PWHT-1 in Figure 1). On the other hand, the second T92/T92 weldment was subjected to its full renormalization consisting of the complete reaustenitization at 1060 °C (i.e., well above the Ac_3_ temperature of T92 steel) for 20 min and subsequently cooled on still air, followed by its conventional subcritical tempering (see the PWHT-2 in Figure 1). From both weldments, twelve cylindrical tensile test specimens of cross-weld (c-w) configuration with partly discontinuous M6 thread (due to the above specified tube wall thickness) within their head portions were machined. A schematic illustration of the tensile test specimen is shown in Figure 2.

**Figure 3:**
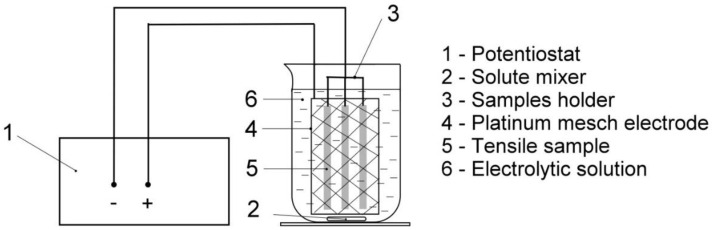
Schematic illustration of electrolytic hydrogenation.

Electrolytic hydrogenation, i.e., cathodic hydrogen charging of prepared cylindrical c-w tensile specimens was performed in electrolytic solution of 1M HCl with 0.1N N_2_H_6_SO_4_ at a current density of 300 A/m^2^. The hydrogenation was realized at room temperature for 24 h. This procedure has been optimized and used in our several former studies [15,30,32] which indicated full saturation of tensile specimens by hydrogen after 24 h of their electrolytic hydrogenation. Similar findings, supported by hydrogen concentration measurements indicated the same or even shorter hydrogenation time for achieving the hydrogen concentration saturation in electrochemically hydrogen-charged alloy steels, as reported in other studies, e.g., [33,34,35]. Yin et al. [36] indicated that the content of diffusible hydrogen tends to be the saturation state when the hydrogen charging time reaches 48 h. However, they showed that the difference in diffusible hydrogen concentration for 24 and 48 h of hydrogen charging was already rather small (i.e., within experimental value scattering). A schematic illustration of the whole experimental setup is visualized in Figure 3.

The room-temperature tensile tests were performed for individual welds’ PWHT states in both hydrogen-free and hydrogen-charged conditions. The tensile testing was carried out using TIRATEST 2300 universal testing machine (TIRA GmbH, Schalkau, Germany) at a crosshead speed of 0.05 mm/min. Three tensile test specimens per each state (i.e., “PWHT-1”, “PWHT-2”, “PWHT-1 + hydrogen”, and “PWHT-2 + hydrogen”) were investigated. The hydrogen-charged samples were tested immediately after the electrolytic hydrogen charging. The evaluation of c-w tensile properties (i.e., yield stress “YS” estimated as 0.2% proof stress, ultimate tensile strength “UTS”, total elongation at fracture “EL”, and reduction of area at fracture “RA”) involved the calculation of their average values and corresponding standard deviations. 

Local mechanical properties of studied weldments were characterized by means of hardness measurements which were performed using a Vickers 432 SVD hardness tester (Wolpert Wilson Instruments, division of Instron Deutschland GmbH, Aachen, Germany) on plain surfaces of longitudinal sections of fractured tensile specimens. This procedure was also helpful for indication of local strain hardening effects within the studied weldments during the tensile tests. The referential, i.e., un-deformed samples corresponding to both initial PWHT states were also tested for hardness. All the hardness measurements were performed at 98 N loading for 10 s per measurement.

Microstructural analyses of the studied weldments were performed on the conventionally prepared metallographic samples (i.e., wet grinding on SiC papers with granularity from 500 to 1200 grit, cloth polishing with a diamond paste suspension of a particle size ranging from 1 to 0.25 μm and final etching in a solution consisting of 120 mL CH_3_COOH, 20 mL HCl, 3 g picric acid, and 144 mL CH_3_OH) using the light optical microscope OLYMPUS GX71 (OLYMPUS Europa Holding GmbH, Hamburg, Germany) and the scanning electron microscope (SEM) JEOL JSM-7000F (Jeol Ltd., Tokyo, Japan). Fractographic analyses were carried out using the SEM Tescan Vega-3 LMU (TESCAN Brno, s.r.o., Czech Republic).

## 3. Results and Discussion

### 3.1. Microstructures

**Figure 4:**
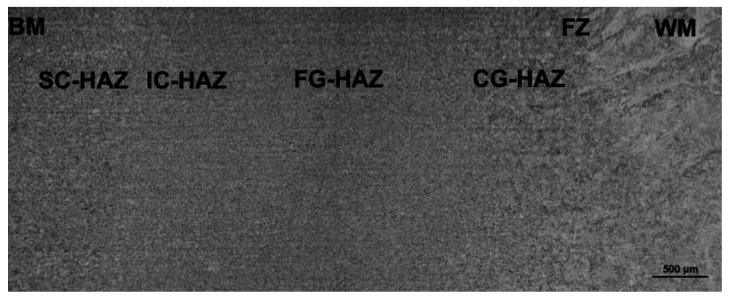
Light-optical micrograph of T92/T92 weldment after the tempering PWHT-1.

Since the qualitative microstructural characteristics of T92/T92 weldments are, in principle, symmetrically distributed with respect to the weld centerline, only one half part of the cross-weld microstructure was documented. Figure 4 shows the light-optical micrograph of T92/T92 weldment in conventionally tempered, i.e., PWHT-1 material state. It can be clearly seen that the weldment after the PWHT-1 exhibits a typical microstructural gradient consisting of individual microstructural sub-regions, i.e., BM, SC-HAZ, IC-HAZ, FG-HAZ, CG-HAZ, FZ, and WM. These microstructural sub-regions are generally formed of tempered martensitic-ferritic structures with different tempering grades of martensite, depending on the reached local peak temperatures (i.e., the temperature gradient) during the welding thermal cycle.

**Figure 5:**
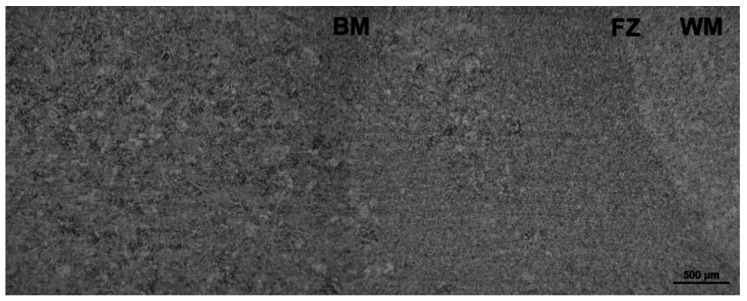
Light-optical micrograph of T92/T92 weldment after the renormalizing-and-tempering PWHT-2.

The microstructural transitions SC-HAZ/IC-HAZ, FG-HAZ/CG-HAZ, and FZ/WM are clear thanks to the observed differences in grain size and morphology. However, the microstructural transitions BM/SC-HAZ and IC-HAZ/FG-HAZ cannot be clearly differentiated by means of light optical microscopy and, thus, they are only roughly estimated in Figure 4. Figure 5 shows the light-optical micrograph of T92/T92 weldment in renormalized-and-tempered, i.e., PWHT-2 material state.

**Figure 6:**
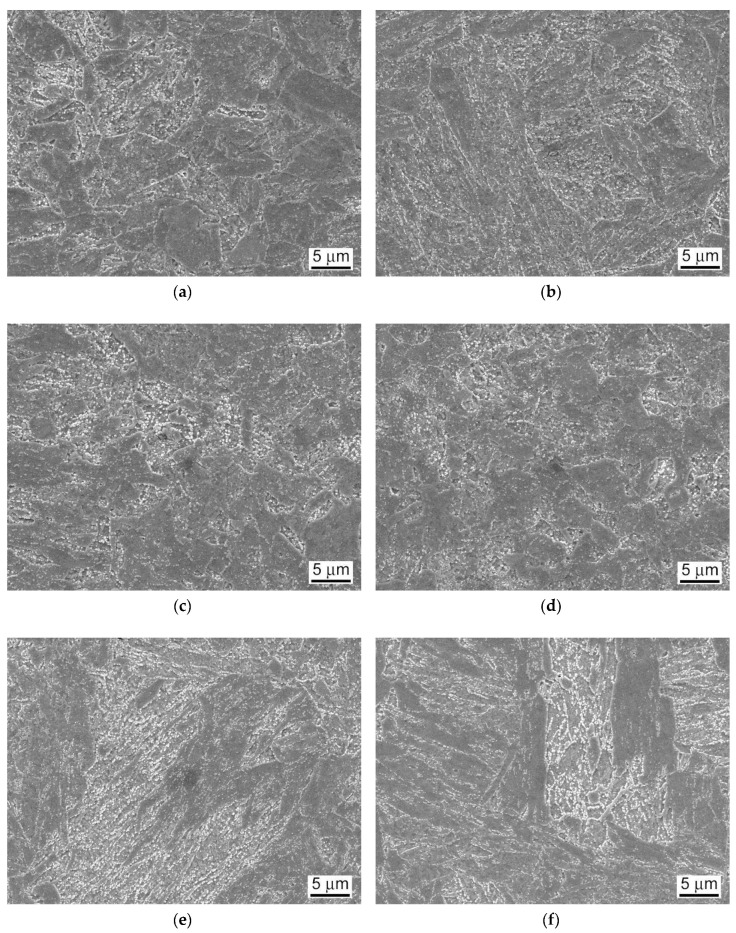
SEM-micrographs of individual microstructural zones of T92/T92 weldment after the tempering PWHT-1: (**a**) BM; (**b**) SC-HAZ; (**c**) IC-HAZ; (**d**) FG-HAZ; (**e**) CG-HAZ; and (**f**) WM.

**Figure 7:**
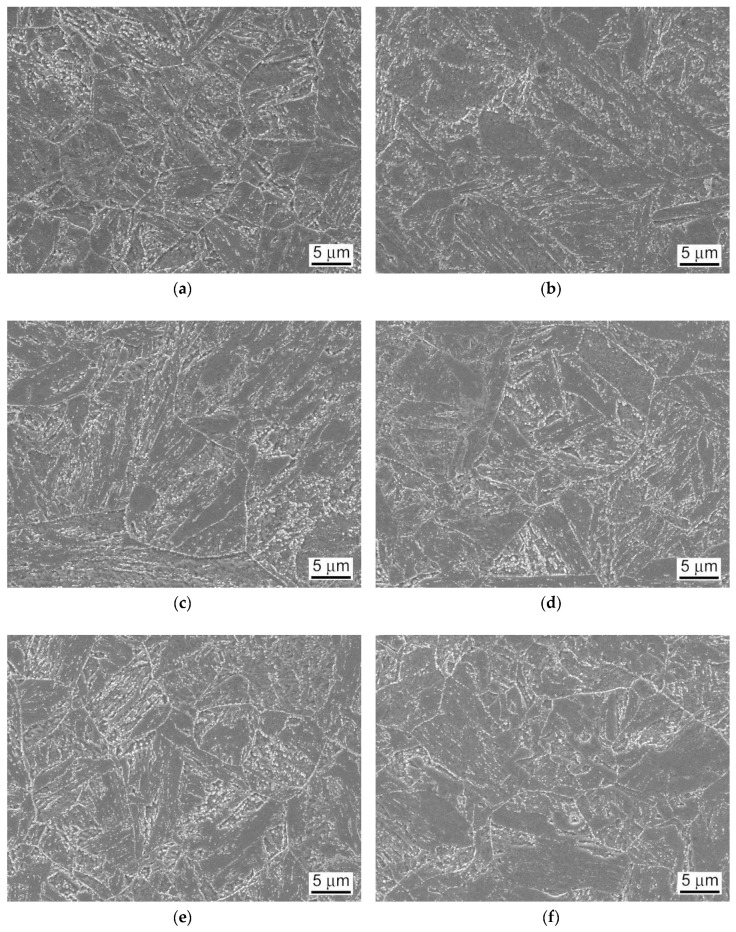
SEM-micrographs of individual microstructural zones of T92/T92 weldment after the renormalizing-and-tempering PWHT-2: (**a**) BM; (**b**) former SC-HAZ; (**c**) former IC-HAZ; (**d**) former FG-HAZ; (**e**) former CG-HAZ; and (**f**) WM.

It can be seen that the weldment after the PWHT-2 shows quite homogenized microstructure as a consequence of the performed renormalization treatment. Within the renormalized-and-tempered weldment, only the regions of BM and WM can be clearly distinguished (Figure 5). This observation can be directly related to the homogenization effect of the applied PWHT-2 resulting in notable suppression of the original T92 HAZ microstructural gradient due to the performed renormalization. However, it should be noted that Figure 5 indicates also a certain microstructural refinement within the former CG-HAZ and WM regions compared to the microstructure of rest BM involving the renormalized-and-tempered regions of former SC-HAZ, IC-HAZ, and FG-HAZ. The detailed SEM-micrographs of individual microstructural zones of T92/T92 weldment after the PWHT-1 and PWHT-2 are shown in Figure 6 and Figure 7, respectively.

The phase composition of normalized and tempered martensitic steels of T/P92 grade is generally known and consists of ferritic matrix and strengthening precipitates of intergranular M_23_C_6_ (M = Cr, Fe…) carbides and intragranular MX (M = V, Nb; X = C, N) carbo-nitrides [37,38,39,40,41]. The same phase composition is to be expected also in the currently studied T92/T92 weldments in both the conventionally tempered and renormalized-and-tempered PWHT conditions. Although predicted by the phase diagram in Figure 1, the precipitation of intermetallic Fe_2_(W,Mo) Laves phase is not to be expected in the currently studied material states (PWHT-1 and PWHT-2) due to insufficient time for its creation related to slow diffusion kinetics of the tungsten and molybdenum atoms in the ferrite solid solution. This assumption has already been evidenced in our several former studies [27,28,29] about the effect of PWHT conditions on microstructure and various properties of dissimilar T92/TP316H ferritic/austenitic weldments for high temperature applications.

By comparison of individual microstructures in Figure 6 and Figure 7, it can be stated that the performed PWHT-2 did not induce full microstructural homogenization of studied weldment with respect to the grain size. Thus, from the observed microstructural characteristics in Figure 7, it cannot be explicitly judged about the appropriateness of the used PWHT-2. Although the original HAZ microstructural gradient has been considerably suppressed, some recognizable microstructural heterogeneity among former HAZ sub-regions is still to be observed. The originally fine-grained regions (IC-HAZ, FG-HAZ) related to PWHT-1 became notably coarse-grained after the PWHT-2. On the contrary, the originally coarse-grained regions (CG-HAZ and WM) became partly refined. The observed microstructural changes can be related to variant (non-uniform) microstructural evolution in individual sub-regions during the PWHT-2 due to pre-existing microstructural differences originated from the primary welding-induced microstructural changes. The IC-HAZ microstructure is formed in the region of BM heated up during the welding to inter-critical peak temperatures (i.e., the temperatures in Ac_1_-Ac_3_ range). Accordingly, the IC-HAZ is formed of fine-grained microstructure consisting of over-tempered (i.e., non-transformed) martensite (i.e., ferrite with coarsened precipitates of original undissolved carbides) and newly formed martensite created on cooling from fine-grained non-saturated austenite. The FG-HAZ microstructure is formed in the region of BM heated up during the welding to peak temperatures just above Ac_3_ up to about 1100 °C. After subsequent cooling, the resulting FG-HAZ consists of newly formed martensite created from fine-grained non-saturated austenite and coarsened precipitates of original undissolved carbides. As shown in Figure 7, after the PWHT-2 both the IC-HAZ and FG-HAZ microstructures exhibit pronounced grain growth which can be related to the lower pinning effect of the coarsened carbide precipitates on the grain boundaries. Thus, the evolution of pronounced grain coarsening in both originally fine-grained IC-HAZ and FG-HAZ microstructures during the renormalization seems to be associated with their low thermal stability in as-welded material condition, enhancing the thermodynamic driving force for the observed microstructural changes. In contrast to the IC-HAZ and FG-HAZ regions, the original CG-HAZ and WM regions are created as a result of on-cooling phase transformations from the highest peak temperatures reached during the welding thermal cycle. Thanks to carbide dissolution at considered peak temperatures, these regions consist of coarse grain structures with the highest level of transformation (i.e., martensitic) hardening. During renormalization at 1060 °C (Figure 1) of these primarily coarse-grained microstructures, thermodynamic conditions for further grain growth are rather unfavorable due to a relatively low renormalization temperature compared to the considered highest peak temperatures. Instead, thanks to the effect of carbon supersaturation in these martensitic microstructures, the creation of newly formed (small) austenite grains on heating to the renormalization temperature is thermodynamically favored. This transformation behavior during the weld renormalization followed by on-cooling phase transformations and final tempering is assumed to be the reason for the creation of partly refined microstructures of CG-HAZ and WM after the PWHT-2.

### 3.2. Mechanical Properties

**Figure 8:**
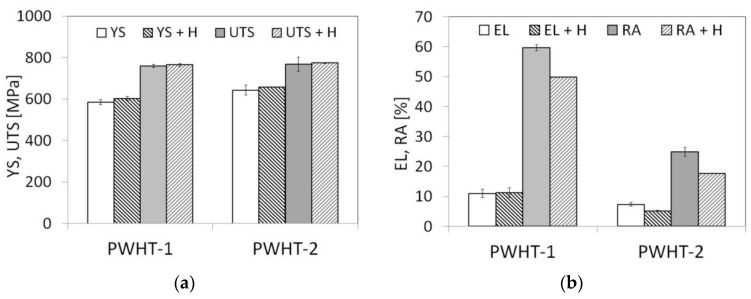
The effects of PWHT conditions and subsequent electrolytic hydrogenation on room-temperature tensile properties of investigated T92/T92 weldments: (**a**) strength properties and (**b**) deformation properties.

The effects of initial PWHT conditions in combination with subsequent electrolytic hydrogenation of the studied T92/T92 weldments on their room-temperature tensile properties are shown in Figure 8.

It can be seen that the renormalizing-and-tempering PWHT-2 of studied T92/T92 weldment resulted only in a small increase of the strength properties, i.e., the YS and UTS values, compared to those of the weldment after the tempering PWHT-1 (Figure 8a). Thus, it can be stated that the effect of various PWHT conditions on the resulting strength properties of studied weldment was rather insignificant. In addition, the differences in measured strength properties between the hydrogen-free and hydrogen-charged weldments in the both PWHT conditions were also quite negligible (Figure 8a). On the contrary, the renormalizing-and-tempering PWHT-2 of studied weldment resulted in a significant decrease of the deformation properties, i.e., the EL and RA values, compared to those of the weldment after the tempering PWHT-1 (Figure 8b). Additional hydrogen charging of the studied weldments in both PWHT material states led to further deterioration of their deformation properties (Figure 8b). However, the observed detrimental effect of the renormalizing-and-tempering PWHT-2 on the deformation properties of the weldments was much more pronounced in comparison with the effect of electrolytic hydrogenation. The measure of individual studied effects (i.e., the heat treatment procedure and electrolytic hydrogenation) on the plasticity deterioration can be quantitatively estimated using the so-called embrittlement index EI:

(1) $$\mathrm{EI}\;(0,\;x)\;=\;\frac{RA_{0}-RA_{x}}{RA_{0}}\;\times \;100\%$$

where *RA*_0_ and *RA_x_* are the values of reduction of area at fracture of two considered material states, and the subscripts “0” and “*x*” refer to the states selected as initial and final, respectively [30]. Thus, the calculated values of the embrittlement index using the average *RA* values (Figure 8b) are summarized in Table 2. From the calculated values of embrittlement index in Table 2, it is clear that the highest degree of embrittlement of studied weldments is caused by the application of renormalizing-and-tempering PWHT-2 (row 1). When comparing the effects of additional hydrogen charging, the higher measure of hydrogen embrittlement is indicated for the weldments processed by the PWHT-2 (row 3) compared to the weldments processed by the PWHT-1 (row 2). 

**Figure 9:**
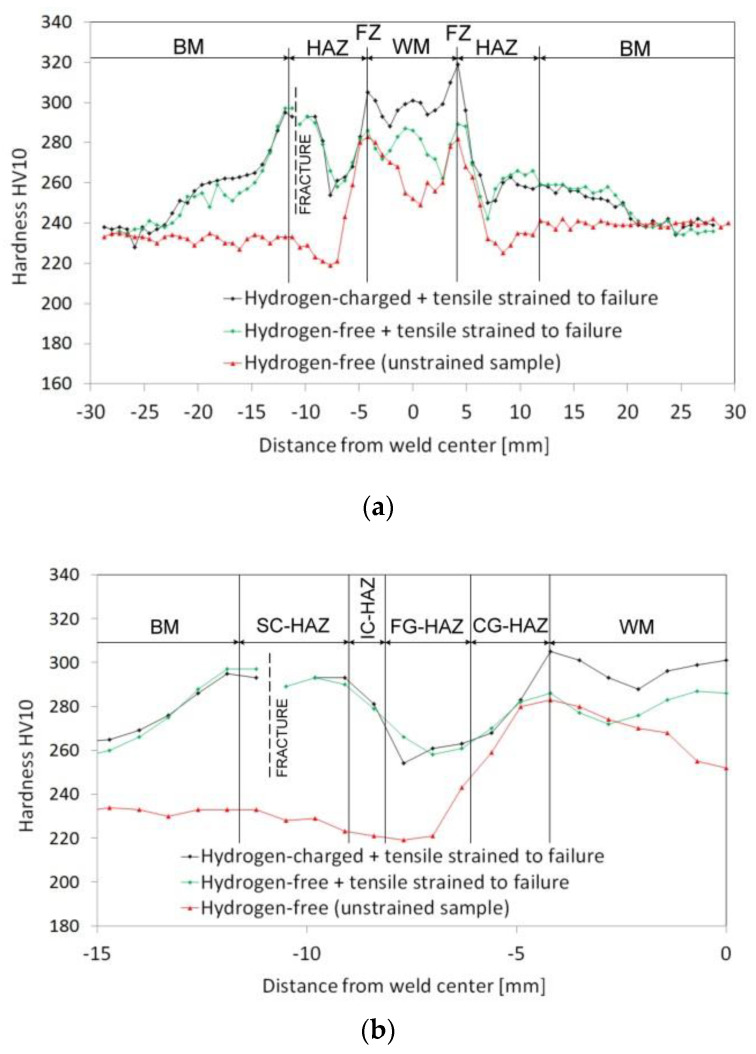
The effects of electrolytic hydrogenation and subsequent room-temperature tensile testing on cross-weld hardness profiles of T92/T92 weldments initially heat treated by conventional tempering PWHT-1: (**a**) overall hardness profiles and (**b**) detailed hardness profiles focused on the HAZ.

**Figure 10:**
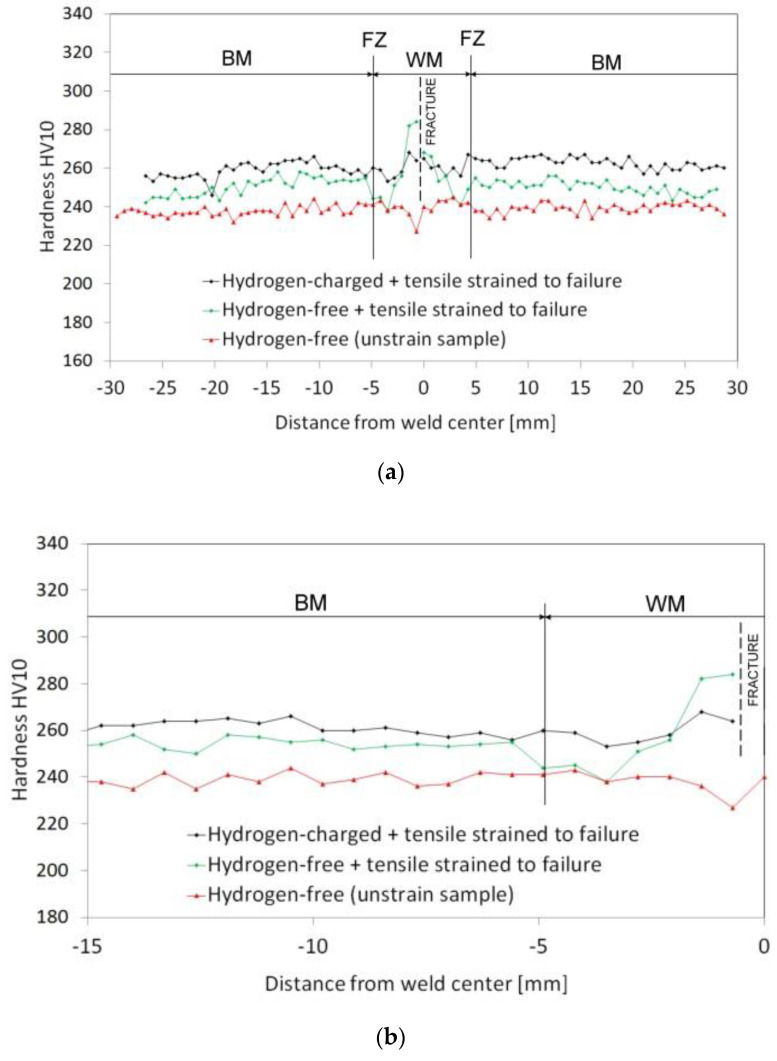
The effects of electrolytic hydrogenation and subsequent room-temperature tensile testing on cross-weld hardness profiles of T92/T92 weldments initially heat treated by renormalizing-and-tempering PWHT-2: (**a**) overall hardness profiles and (**b**) detailed hardness profiles focused on the former HAZ.

The obtained findings about the highly detrimental effect of the welds’ homogenization treatment on their plastic properties can be explained by considering the microstructural changes induced by the renormalizing-and-tempering PWHT-2. The observed microstructural differences between the weldments in PWHT-1 and PWHT-2 material states (Figure 4, Figure 5, Figure 6 and Figure 7) have crucial effects on the localization of plastic deformation during the tensile testing. In order to indicate local strain hardening behavior in the studied T92/T92 weldments during the tensile straining, c-w Vickers hardness measurements were carried out on the plain surfaces of longitudinal sections of fractured tensile samples after the tensile tests. Figure 9 and Figure 10 show the c-w Vickers hardness profiles of the studied weldments initially heat treated using the PWHT-1 and PWHT-2, respectively. Visible interruptions within the c-w hardness profiles indicate the fracture locations of broken tensile test specimens after the room-temperature tensile tests. The c-w hardness profiles of the referential unstrained samples are also included for comparison. Significant differences between the c-w hardness profiles of T92/T92 weldments in various initial PWHT conditions are clearly visible when comparing Figure 9 and Figure 10. By comparison of both unstrained samples (i.e., in PWHT-1 and PWHT-2 conditions), it can be concluded that the weldment after the tempering PWHT-1 shows a steep hardness gradient in its HAZ. The highest hardness values are measured in the WM and FZ regions which can be related to the highest measure of transformation (i.e., martensitic) hardening in these locations. On the other hand, the lowest hardness values are typically measured in the FG-HAZ, IC-HAZ, and partly SC-HAZ regions which are known to be locations with the greatest degradation of transformation hardening during the welding thermal cycle [10].

The hardness profiles of both the hydrogen-free and hydrogen-charged specimens show the strain-induced hardness peaks in the formerly soft (i.e., in the initial unstrained state) SC-HAZ in the location of tensile test fracture. Thus, the originally softer areas within the T92/T92 weldment in the PWHT-1 material state represent the locations of the localization of the highest plastic deformation during tensile testing. 

**Figure 11:**
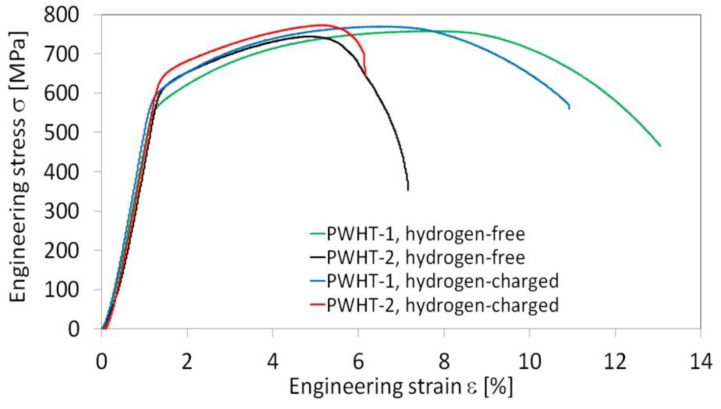
The effects of PWHT conditions and subsequent electrolytic hydrogenation on tensile deformation behavior of investigated T92/T92 weldments.

The necking-related hardness peaks at fracture locations are the result of strain hardening due to the localization of plastic deformation after reaching plastic instability during tensile testing. This can be supported by the fact that the more ductile samples after the PWHT-1 (in both hydrogen-free and hydrogen-charged conditions, see Figure 9) show significant hardness peaks, compared to the less pronounced hardness peak related to the embrittled sample after the PWHT-2. Moreover, the hydrogenated sample after the PWHT-2 shows almost total suppression of its hardness peak in fracture location (see Figure 10). The localized plastic deformation can reasonably explain the better deformation properties of the highly heterogeneous T92/T92 weldment in the PWHT-1 material state, compared to the homogenized weldment in the PWHT-2 material state. The localized plastic deformation can be quantified by true fracture strain ε*_f_*, as it follows:

(2) $$\varepsilon_{f}=ln\frac{A_{0}}{A_{f}}$$

where *A*_0_ and *A_f_* are the values of original cross-sectional area and final (i.e., minimal) cross-sectional area at the point of fracture of the tensile test specimen. The calculated ε*_f_* values indicating the level of accumulated plastic deformation at the location of final fracture are shown in Table 3:

In our case, the reason to cause localized plasticity is related to the tri-axial stress state (i.e., the stress triaxiality) induced by plastic instability at local microstructural heterogeneities (microstructural notches), namely the HAZ microstructural gradient and WM microstructural heterogeneity in the PWHT-1 and PWHT-2 material states, respectively. For the sake of completeness, it should be noted that for the weldments in PWHT-1 material state, the increased strain hardening effects were observed not only at the final fracture locations but also at the locations of the concurrent necking areas (Figure 9a). The reason for this behavior was to be expected since the c-w configuration of investigated tensile samples implied symmetrical occurrence of equivalent HAZ microstructural gradients at the both sides of welded base materials. The undeformed weldment after the renormalizing-and-tempering PWHT-2 shows equalized course of hardness values as a result of the weld microstructure homogenization (Figure 10). Moreover, the hardness profile of hydrogen-free weldment, initially subjected to the renormalizing-and-tempering PWHT-2, shows after the tensile test the localized strain hardening at the location of final failure in WM. The hydrogen-charged weldment shows the same failure location but without the occurrence of significant strain hardening at fracture location. The observed WM failures occurred likely due to the fact that the WMs represent the critical microstructural regions even after the performed homogenization treatment due to their original as-cast microstructure and thus higher impurity content and inhomogeneity compared to the BM. For comparison, Figure 11 shows four selected engineering stress-strain curves representing the overall tensile deformation behavior of the studied T92/T92 weldments in their individual material states with respect to the used PWHT conditions and hydrogen charging application. From the obtained results, it can be concluded that the tensile deformation and fracture behavior of studied T92/T92 weldments is more sensitive to the initial PWHT conditions than to the applied electrolytic hydrogenation. In spite of the occurrence of certain hydrogen embrittlement in the studied weldments, it has been shown that the thermal deterioration caused by the use of renormalizing-and-tempering PWHT-2 is much more significant. This statement is additionally supported by the fact that the final failure locations are primarily pre-determined by the used PWHT conditions, i.e., regardless of hydrogen charging application. The T92/T92 weldments after the tempering PWHT-1 were always broken in their SC-HAZs, whereas the weldments after the renormalizing-and-tempering PWHT-2 always fractured in their WMs.

**Figure 12:**
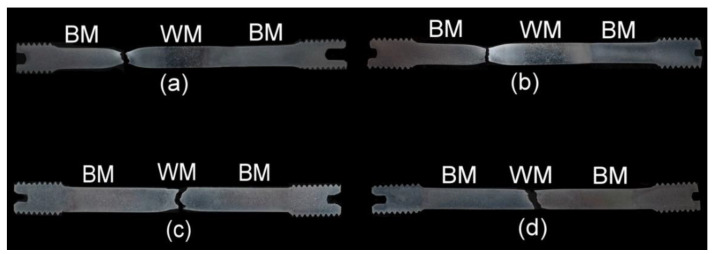
Photo-macrographs of fractured tensile samples’ counterparts indicating various failure locations after room-temperature tensile tests of T92/T92 weldments in their individual material states: (**a**) “PWHT-1”; (**b**) “PWHT-1 + hydrogen”; (**c**) “PWHT-2”; (**d**) “PWHT-2 + hydrogen”.

Figure 12 shows representative photo-macrographs of longitudinal sections of the fractured tensile samples’ counterparts showing two different failure locations of the studied T92/T92 weldments in their all investigated material states.

The detailed discussion on mutual correlation between the fractographic and microstructural characteristics of studied T92/T92 weldments will be provided within the following section.

### 3.3. Fractography

**Figure 13:**
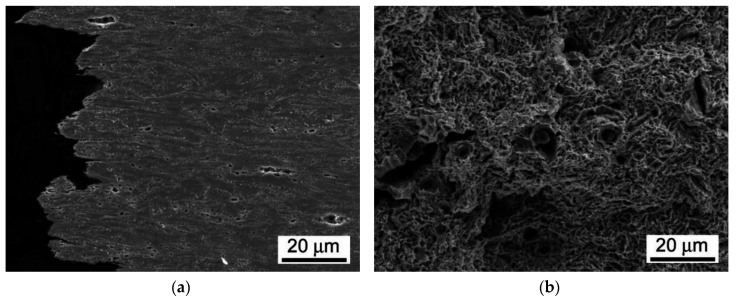
Fracture analysis of hydrogen-free T92/T92 weldment initially processed by the PWHT-1 and subsequently ruptured in SC-HAZ after the room-temperature tensile test: (**a**) fracture path and microstructure beneath the fracture; (**b**) fracture surface showing pure ductile dimple tearing.

**Figure 14:**
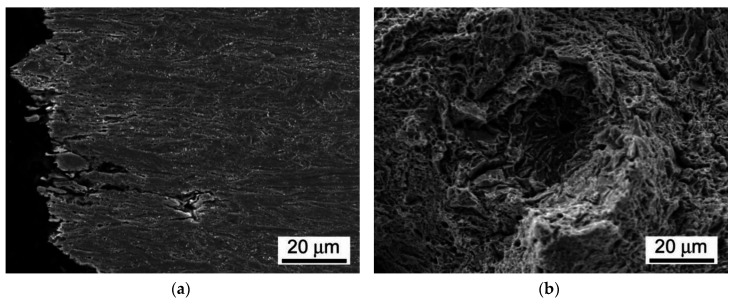
Fracture analysis of hydrogen-charged T92/T92 weldment initially processed by the PWHT-1 and subsequently ruptured in SC-HAZ after the room-temperature tensile test: (**a**) fracture path and microstructure beneath the fracture; (**b**) fracture surface showing mixed fracture consisting of both transgranular quasi-cleavage with a “fish-eye” morphology and ductile dimple tearing.

As already discussed, and shown in Figure 12, the room-temperature fracture behavior of studied T92/T92 weldments during tensile straining is primarily controlled by the used PWHT conditions, whereas the effect of electrolytic hydrogenation is less critical. The observation of macroscopically ductile failures (Figure 12a,b) within the SC-HAZs of both the hydrogen-free and hydrogen-charged T92/T92 weldments after the tempering PWHT-1 and tensile straining, can be related to higher deformability of the over-tempered SC-HAZs compared to the BM and WM regions. The corresponding fracture analyses for the hydrogen-free and hydrogen-charged weldments initially processed by the PWHT-1 are shown in Figure 13 and Figure 14, respectively. Although the both hydrogen-free and hydrogen-charged weldments in PWHT-1 material state were broken in a recognizably ductile manner in their SC-HAZs (see the fracture paths in Figure 13a and Figure 14a), the signs of hydrogen embrittlement are clearly visible for the hydrogen-charged weldment. In Figure 13a and Figure 14a the SC-HAZ microstructures beneath final fractures are characterized by intensive deformation of grains in a direction of dominating tensile stresses. The microstructure of hydrogen-free weldment after the PWHT-1 (Figure 13a) shows randomly distributed precipitates and signs of microvoid coalescence along the elongated grain boundaries indicating the origin of the formation of ductile dimples on the fracture surface (Figure 13b). Whereas the fracture surface of hydrogen-free T92/T92 weldment in PWHT-1 material state shows completely ductile dimple fracture (Figure 13b), the fracture surface of the hydrogen-charged weldment shows quasi-cleavage areas with typical “fish-eye” morphology due to the local hydrogen-embrittlement beside ductile dimple tearing areas (Figure 14b). The hydrogenated sample after the PWHT-1 shows in its microstructure beneath the fracture surface hydrogen-assisted cracks and even some grain fragmentation (Figure 14a). The “fish-eye” fractographic object on the fracture surface (Figure 14b) represents the hydrogen embrittled quasi-cleavage zone created radially along the central inclusion.

**Figure 15:**
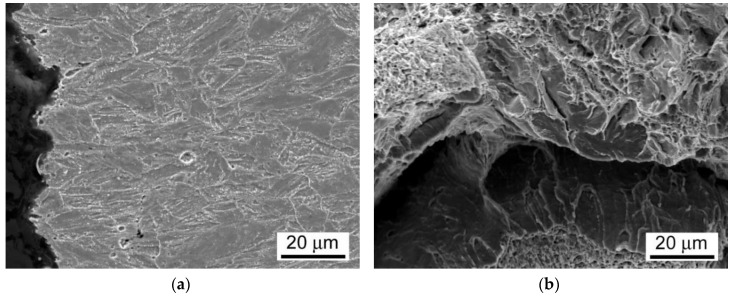
Fracture analysis of hydrogen-free T92/T92 weldment initially processed by the PWHT-2 and subsequently ruptured in WM after the room-temperature tensile test: (**a**) fracture path and microstructure beneath the fracture; (**b**) fracture surface showing mixed fracture consisting of both ductile dimple tearing and transgranular quasi-cleavage.

**Figure 16:**
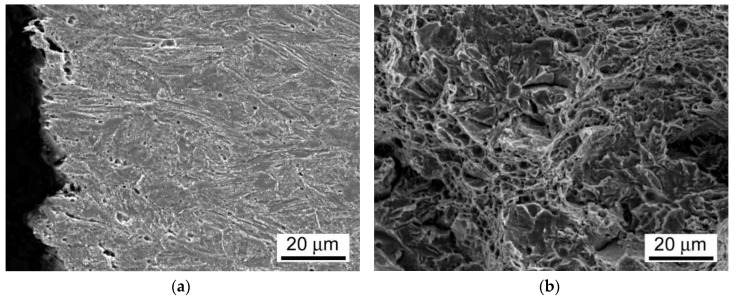
Fracture analysis of hydrogen-charged T92/T92 weldment initially processed by the PWHT-2 and subsequently ruptured in WM after the room-temperature tensile test: (**a**) fracture path and microstructure beneath the fracture; (**b**) fracture surface showing mixed fracture consisting of transgranular quasi-cleavage with a “fish-eye” morphology besides ductile dimple ridges and some inter-lath decohesion areas.

On the other hand, the observation of rather brittle failures (Figure 12c,d) within the WMs of both the hydrogen-free and hydrogen-charged T92/T92 weldments after the renormalizing-and-tempering PWHT-2 can be related to their lower deformability. It appears as a result of thermal embrittlement and possibly remaining microstructural heterogeneities of the original as-cast WMs microstructures (e.g., dendritic segregation) due to insufficient homogenization (i.e., short time for the alloying elements redistribution during performed reaustenitization, see Figure 1). Thus, the WMs represent the most heterogeneous regions within the renormalized-and-tempered weldments. This finding indicates the reason for their high propensity for the localization of plastic deformation and final failure occurrence during tensile straining. The corresponding fracture analyses for the hydrogen-free and hydrogen-charged weldments initially processed by the PWHT-2 are shown in Figure 15 and Figure 16, respectively.

The fracture paths in Figure 15a and Figure 16a indicate lower ductility failures for both the hydrogen-free and hydrogen-charged T92/T92 weldments after the renormalizing-and-tempering PWHT-2 compared to the weldments after the tempering PWHT-1 (Figure 13a and Figure 14a). The clearer differences between the fracture mechanisms related to the hydrogen-free and hydrogen-charged weldments after the PWHT-2 are distinguishable on the fracture surfaces in Figure 15b and Figure 16b. Whereas the fracture surface of hydrogen-free weldment exhibits mixed fracture mechanisms including the ductile dimple tearing and transgranular quasi-cleavage (Figure 15b), the fracture surface of hydrogenated weldment shows even more complex fracture mechanisms involving transgranular quasi-cleavage with a “fish-eye” morphology besides ductile dimple tearing and some inter-lath decohesion (Figure 16b). The reason for the observed fracture morphology changes can be explained by considering local microstructural characteristics beneath the fracture surfaces. In Figure 15a and Figure 16a the WM microstructures beneath final fractures are characterized by polygonal grains and sub-grains almost continuously decorated with coarsened secondary phase precipitates indicating thermal embrittlement induced by PWHT-2. That is why the both hydrogen-free and hydrogen-charged samples show mixed fractures consisting of both ductile dimple tearing and brittle transgranular quasi-cleavage (Figure 15b and Figure 16b). The reason for the additional occurrence of “fish-eye” fracture morphology on the fracture surface of the hydrogenated sample treated by PWHT-2 (Figure 16b) is basically the same as in the case of hydrogenated sample after the PWHT-1. However, the higher level of thermal embrittlement after the PWHT-2 was at the same time the reason for higher grade of hydrogen embrittlement due to the existence of more nucleation sites for the creation of “fish-eye” fractographic objects. 

As already shown in previous section, the hardness profile of undeformed c-w sample of T92/T92 weldment after the PWHT-2 (Figure 10) showed somewhat lower hardness in WM compared to the rest BM portions. This observation may also be related to lower alloying of WM compared to BM. Thus, the preferential deformation of softer WMs during room-temperature tensile deformation of the welds after the PWHT-2 gave rise to their higher propensity for localization of plastic deformation and final failure occurrence. It can be concluded that the use of renormalizing-and-tempering PWHT-2 was found to be rather inconvenient for improving the microstructure and mechanical properties of the investigated T92/T92 weldments. However, based on the results obtained from the cross-weld tensile tests, the hydrogen embrittlement susceptibility of the investigated weldments was rather low for both the applied PWHT conditions.

## 4. Conclusions

In present study the T92/T92 weldments in two different initial PWHT states (i.e., in either conventionally tempered PWHT-1 state or in renormalized-and-tempered PWHT-2 state) were investigated in terms of the electrolytic hydrogenation effect on their room-temperature tensile properties and fracture behavior. Here are the main conclusions:

- The observed microstructural variations of studied weldments induced by different initial PWHT conditions had crucial effects on their resulting tensile deformation and fracture behavior in both the hydrogen-free and hydrogen-charged conditions. The weldments after the tempering PWHT-1 exhibited a typical HAZ microstructural gradient consisting of the CG-HAZ, FG-HAZ, IC-HAZ, and SC-HAZ, whereas the weldments after the renormalizing-and-tempering PWHT-2 showed quite homogenized microstructure of former HAZ. In addition, the regions of WM and former CG-HAZ showed certain microstructural refinement compared to the rest of the BM.
- The effects of both the PWHT conditions applied and electrolytic hydrogenation on the resulting strength properties, i.e., the YS and UTS values of studied weldments, were rather insignificant. A small increase of the measured strength properties has been observed after the renormalizing-and-tempering PWHT-2 compared to the tempering PWHT-1, which was reasonably attributed to the overall suppression of the former HAZ microstructural gradient with its soft microstructural sub-regions as indicated by c-w hardness measurements. As expected, some small hydrogen-induced hardening effects were observed for the weldments in both the PWHT-1 and PWHT-2 states.
- The used renormalizing-and-tempering PWHT-2 of studied weldment resulted in a significant decrease of its plastic properties, i.e., the EL and RA values, compared to those of the weldment after the conventional tempering PWHT-1. Thus, the use of renormalizing-and-tempering PWHT-2 was found to be quite inappropriate for improving the microstructure and mechanical properties of the investigated T92/T92 weldments. The application of electrolytic hydrogenation of the studied weldments in their both PWHT conditions led to additional detrimental effects of their plastic properties. Although the results indicated recognizably higher hydrogen embrittlement susceptibility for the renormalized-and-tempered weldments compared to the conventionally tempered ones, it can be concluded that all studied weldments show sufficient resistance against hydrogen embrittlement in conditions of the present investigation.

## Figures and Tables

**Table 1:** Chemical composition (wt.%) of T92 base material (T92 BM) and T92-based filler metal (T92 FM) used for fabrication of T92/T92 weldments.

Material	C	N	Si	Mn	Cr	Mo	W	B	Ni	Ti	V	Nb	Fe
T92 BM	0.11	0.05	0.38	0.49	9.08	0.31	1.57	0.002	0.33	-	0.2	0.07	rest
T92 FM	0.11	0.05	0.2	0.6	8.8	0.5	1.6	-	0.7	-	0.2	0.05	rest

**Table 2:** Embrittlement index for individual material states.

Row	0	*x*	EI (0, *x*) [%]
1	PWHT-1	PWHT-2	58.2
2	PWHT-1	PWHT-1 + H	16.1
3	PWHT-2	PWHT-2 + H	28.9

**Table 3:** Calculated values of true fracture strain for studied weldment in individual material states.

Material State	ε*_f_* (–)
PWHT-1	0.907
PWHT-1 + hydrogen	0.693
PWHT-2	0.286
PWHT-2 + hydrogen	0.195
